# Supplementary material for: A Likelihood Approach for Real-Time Calibration of Stochastic Compartmental Epidemic Models
Source: PLoS Comput Biol. 2017 Jan 17;13(1):e1005257. doi: 10.1371/journal.pcbi.1005257 (PMC5240920; doi:10.1371/journal.pcbi.1005257)
Supplement: S1 File — (TAR.GZ) [file pcbi.1005257.s014.tar.gz › HSPH_Online-SI-Revision/IPoi09/n10-mild/n10-mild_BENCH_table4.pdf]

1	R0	{1.25046, 6.80278}	{1.69981, 11.6438}	{{1.68954, 11.6019}, {1.72065, 11.7288}}	{35.9345, 71.162}	inf	141.	650.094	{415., 903.}	361.06	0.643575	newly	135.	366.849	{216., 539.}	172.187	0.410374	Reff	0.976984	0.774162	{0.607045, 1.09753}	27.6572	ar	3516.	6890.13	{6668., 7124.}	95.9651
2	R0	{1.32013, 4.45847}	{2.0968, 12.1899}	{{2.0968, 12.1899}, {2.0968, 12.1899}}	{58.8328, 173.41}	inf	141.	973.25	{488., 1577.}	590.248	0.800387	newly	183.	497.827	{235., 850.}	172.037	0.388061	Reff	0.815972	0.573043	{0.447876, 0.835575}	31.863	ar	4420.	8222.62	{8121., 8356.}	86.032
3	R0	{1.31785, 5.36796}	{2.15677, 10.1363}	{{2.15677, 10.1363}, {2.15677, 10.1363}}	{63.6582, 88.8296}	inf	276.	578.308	{261., 1131.}	112.723	0.281799	newly	309.	335.385	{153., 631.}	42.6064	0.168942	Reff	0.782671	0.445974	{0.375278, 0.57672}	43.4493	ar	4942.	8360.54	{8266., 8458.}	69.1732
4	R0	{1.38801, 6.16031}	{2.44607, 18.5461}	{{2.35866, 16.9062}, {2.46477, 18.9032}}	{76.2282, 201.058}	inf	192.	1565.07	{1004., 2202.}	716.402	0.893869	newly	196.	558.746	{355., 781.}	187.161	0.445034	Reff	0.811292	0.692408	{0.417955, 1.23961}	41.7679	ar	4790.	8854.37	{8715., 8966.}	84.8512
5	R0	{1.27955, 11.57}	{1.64805, 19.8679}	{{1.6411, 19.8325}, {1.65054, 19.8898}}	{28.7989, 71.7189}	inf	157.	635.717	{403., 888.}	304.916	0.583377	newly	94.	218.804	{129., 304.}	134.436	0.349305	Reff	1.02351	0.83292	{0.647342, 1.17897}	26.1491	ar	3460.	6668.63	{6524., 6822.}	92.735
6	R0	{1.22184, 4.34395}	{1.78545, 6.62306}	{{1.78724, 6.63253}, {1.78724, 6.63253}}	{46.1277, 52.4663}	inf	121.	276.122	{115., 438.}	134.005	0.314264	newly	169.	233.805	{98., 391.}	61.8127	0.197348	Reff	0.87777	0.544584	{0.480589, 0.596759}	37.9582	ar	3524.	7253.78	{7067., 7489.}	105.839
7	R0	{1.23856, 4.59068}	{1.86359, 9.39916}	{{1.85533, 9.28761}, {1.88096, 9.64595}}	{50.4643, 104.744}	inf	125.	556.51	{234., 950.}	345.241	0.591021	newly	186.	366.327	{144., 696.}	106.956	0.285309	Reff	0.844822	0.595474	{0.487921, 0.757717}	34.2478	ar	3855.	7566.96	{7402., 7735.}	96.2895
8	R0	{1.34531, 10.6689}	{1.98338, 15.901}	{{1.97176, 15.6958}, {2.00049, 16.2766}}	{47.4293, 49.041}	inf	364.	1023.62	{657., 1399.}	181.213	0.430613	newly	224.	421.383	{255., 615.}	88.9955	0.256241	Reff	0.92221	0.681895	{0.519727, 0.937178}	31.2919	ar	4852.	7931.33	{7811., 8084.}	63.4652
9	R0	{1.31775, 5.19226}	{2.64628, 19.7701}	{{2.64373, 19.7855}, {2.66225, 19.9254}}	{100.818, 280.761}	inf	255.	1911.51	{1183., 2509.}	649.611	0.858818	newly	288.	641.791	{407., 863.}	122.844	0.33077	Reff	0.778658	0.737438	{0.385456, 1.30424}	42.6388	ar	4708.	9106.57	{9046., 9165.}	93.4275
10	R0	{1.25916, 6.89207}	{1.67732, 9.44073}	{{1.67732, 9.44073}, {1.67732, 9.44073}}	{33.2094, 36.9796}	inf	145.	314.026	{132., 587.}	121.615	0.303993	newly	135.	203.184	{84., 367.}	67.076	0.207207	Reff	0.888841	0.616892	{0.544123, 0.697094}	31.1112	ar	3823.	6805.08	{6638., 6961.}	78.0036
11	R0	{1.20377, 5.46566}	{1.67405, 7.28757}	{{1.67405, 7.28757}, {1.67405, 7.28757}}	{39.0673, 33.3338}	inf	117.	275.816	{131., 511.}	136.73	0.330389	newly	147.	218.	{106., 400.}	60.3652	0.184737	Reff	0.911254	0.590495	{0.528832, 0.661417}	35.1998	ar	3114.	6831.58	{6645., 7060.}	119.383
12	R0	{1.27878, 2.55804}	{2.05801, 8.77121}	{{2.03307, 8.59564}, {2.06386, 8.81239}}	{60.9354, 242.888}	inf	51.	563.738	{227., 1222.}	1005.37	0.949551	newly	100.	380.721	{141., 818.}	281.213	0.471394	Reff	0.804992	0.502958	{0.402659, 0.627826}	40.7268	ar	3930.	8135.39	{8038., 8250.}	107.007
13	R0	{1.41227, 5.45696}	{2.30371, 19.6625}	{{2.30371, 19.6625}, {2.30371, 19.6625}}	{63.1211, 260.32}	inf	198.	1498.42	{984., 2011.}	660.909	0.869823	newly	224.	497.818	{327., 720.}	128.555	0.371838	Reff	0.81262	0.711951	{0.467653, 1.00211}	33.7534	ar	4822.	8636.65	{8547., 8714.}	79.1094
14	R0	{1.32917, 7.27593}	{2.22772, 17.5402}	{{2.21213, 17.2871}, {2.23964, 17.6755}}	{67.602, 141.072}	inf	261.	1118.35	{583., 1732.}	328.486	0.597051	newly	225.	417.029	{207., 671.}	90.2544	0.255519	Reff	0.839903	0.628845	{0.420968, 1.04076}	41.7308	ar	4758.	8499.7	{8388., 8608.}	78.6401
15	R0	{1.31629, 9.97481}	{1.79072, 11.5505}	{{1.79692, 11.6747}, {1.79692, 11.6747}}	{36.043, 16.2701}	inf	321.	596.545	{278., 1015.}	92.4384	0.259371	newly	226.	326.114	{152., 554.}	59.5837	0.193451	Reff	0.982216	0.644236	{0.543568, 0.782738}	35.0285	ar	4543.	7255.89	{6976., 7447.}	59.7157
16	R0	{1.36735, 5.74166}	{2.14466, 13.8098}	{{2.1255, 13.6009}, {2.2143, 14.4309}}	{56.8482, 140.52}	inf	216.	1156.57	{591., 1620.}	435.449	0.702924	newly	224.	533.985	{276., 803.}	139.512	0.352513	Reff	0.874557	0.607539	{0.439308, 0.790282}	32.9138	ar	4516.	8331.72	{8203., 8458.}	84.4934
17	R0	{1.27524, 13.2396}	{1.58037, 12.9004}	{{1.5767, 12.7642}, {1.58973, 13.3174}}	{23.9276, 2.80888}	inf	156.	573.487	{214., 827.}	271.565	0.540443	newly	82.	317.41	{111., 427.}	290.588	0.563852	Reff	1.15027	1.1737	{0.865924, 1.47879}	15.7421	ar	3731.	6302.82	{6098., 6456.}	68.9311
18	R0	{1.21884, 7.71545}	{1.6568, 10.5289}	{{1.6568, 10.5289}, {1.6568, 10.5289}}	{35.9325, 36.4651}	inf	198.	485.514	{214., 852.}	148.458	0.35734	newly	194.	299.	{128., 562.}	67.7487	0.207766	Reff	0.931559	0.709863	{0.571265, 0.90892}	27.7113	ar	3080.	6716.49	{6572., 6880.}	118.068
19	R0	{1.34879, 6.71464}	{2.00785, 10.5661}	{{1.96658, 10.2409}, {2.02235, 10.6804}}	{48.863, 57.3596}	inf	164.	446.621	{179., 908.}	172.939	0.356933	newly	162.	253.655	{105., 524.}	75.0745	0.208935	Reff	0.798079	0.479685	{0.412964, 0.602054}	40.1989	ar	4585.	8007.31	{7899., 8139.}	74.6414
20	R0	{1.37284, 5.75036}	{1.88096, 9.64595}	{{1.88096, 9.64595}, {1.88096, 9.64595}}	{37.0123, 67.7451}	inf	225.	589.773	{289., 1030.}	164.04	0.376523	newly	244.	378.568	{183., 685.}	70.2776	0.211806	Reff	0.87848	0.582918	{0.473626, 0.734891}	35.8941	ar	4750.	7634.68	{7468., 7775.}	60.7301
21	R0	{1.29938, 5.36419}	{2.118, 12.1706}	{{2.0968, 12.1677}, {2.12117, 12.1899}}	{63.001, 126.886}	inf	178.	891.667	{478., 1449.}	400.936	0.661484	newly	209.	457.593	{221., 758.}	121.141	0.30536	Reff	0.808604	0.550621	{0.42084, 0.749409}	35.5728	ar	4337.	8259.17	{8167., 8368.}	90.435
22	R0	{1.31882, 7.47626}	{1.89815, 13.1905}	{{1.86902, 12.6353}, {1.94617, 14.0718}}	{43.9281, 76.4312}	inf	191.	786.5	{326., 1207.}	311.78	0.577743	newly	169.	385.121	{170., 629.}	131.024	0.333661	Reff	0.929504	0.668487	{0.515663, 0.896195}	34.2818	ar	3977.	7664.17	{7472., 7888.}	92.7124
23	R0	{1.26215, 3.46375}	{1.95698, 9.80394}	{{1.95791, 9.82151}, {1.95791, 9.82151}}	{55.0512, 183.044}	inf	114.	569.556	{261., 898.}	399.61	0.653555	newly	207.	351.889	{155., 555.}	80.2147	0.231345	Reff	0.848291	0.572094	{0.455997, 0.629468}	40.4771	ar	3607.	7862.2	{7734., 7999.}	117.971
24	R0	{1.16712, 4.19464}	{1.63746, 8.23985}	{{1.62191, 8.12461}, {1.71337, 8.80249}}	{40.299, 96.4376}	inf	82.	170.	{57., 379.}	126.413	0.275678	newly	105.	126.39	{37., 267.}	77.1893	0.285538	Reff	0.873473	0.602153	{0.535771, 0.685744}	32.155	ar	3000.	6618.22	{6327., 6945.}	120.607
25	R0	{1.22751, 3.34517}	{1.73655, 7.10164}	{{1.7356, 7.10024}, {1.739, 7.10524}}	{41.4695, 112.295}	inf	114.	632.815	{314., 987.}	455.101	0.705734	newly	241.	548.037	{252., 921.}	129.599	0.320285	Reff	0.932662	0.70949	{0.552615, 0.911584}	28.7323	ar	3086.	7086.54	{6908., 7222.}	129.635
26	R0	{1.18747, 5.88083}	{1.76659, 10.896}	{{1.76659, 10.896}, {1.76659, 10.896}}	{48.7692, 85.28}	inf	107.	323.711	{171., 704.}	203.665	0.425547	newly	102.	184.658	{97., 398.}	86.6099	0.224223	Reff	0.802017	0.570195	{0.496412, 0.663178}	30.9755	ar	3614.	7210.05	{7006., 7377.}	99.5034
27	R0	{1.29023, 4.76202}	{2.44197, 19.0672}	{{2.43839, 19.0531}, {2.43839, 19.0531}}	{89.2659, 300.402}	inf	148.	1553.1	{898., 2187.}	949.393	0.991729	newly	192.	541.186	{301., 777.}	183.333	0.42907	Reff	0.787814	0.740188	{0.408918, 1.11873}	41.6036	ar	4245.	8843.95	{8751., 8914.}	108.338
28	R0	{1.2258, 12.9056}	{1.6411, 19.8325}	{{1.6411, 19.8325}, {1.6411, 19.8325}}	{33.8799, 53.6736}	inf	171.	412.946	{139., 804.}	147.748	0.343845	newly	91.	137.27	{45., 270.}	76.0915	0.245638	Reff	0.929156	0.677588	{0.572416, 0.834335}	27.821	ar	3639.	6654.59	{6451., 6871.}	82.8688
29	R0	{1.34702, 6.29049}	{2.10521, 13.3892}	{{2.08003, 13.1443}, {2.2143, 14.4309}}	{56.2868, 112.848}	inf	220.	941.924	{498., 1576.}	328.147	0.59388	newly	234.	450.106	{232., 823.}	97.3258	0.266537	Reff	0.838924	0.595952	{0.43535, 0.950366}	38.3745	ar	4560.	8240.73	{8096., 8434.}	80.7177
30	R0	{1.37931, 6.78741}	{2.16285, 14.7752}	{{2.12155, 14.3165}, {2.16755, 14.8261}}	{56.8064, 117.685}	inf	216.	824.148	{401., 1297.}	281.55	0.537971	newly	206.	350.389	{152., 559.}	80.2499	0.237266	Reff	0.813793	0.496363	{0.399263, 0.611032}	39.646	ar	4667.	8373.67	{8265., 8473.}	79.4229
31	R0	{1.16062, 4.47529}	{1.69756, 6.15907}	{{1.69756, 6.15907}, {1.69756, 6.15907}}	{46.2632, 37.6239}	inf	144.	371.9	{119., 747.}	164.861	0.353031	newly	207.	355.1	{111., 740.}	95.9179	0.26915	Reff	0.868956	0.622792	{0.530827, 0.738778}	29.3868	ar	3037.	6932.93	{6787., 7053.}	128.282
32	R0	{1.37647, 18.7122}	{1.73196, 19.1609}	{{1.73196, 19.1609}, {1.73196, 19.1609}}	{25.8262, 2.3979}	inf	353.	320.368	{150., 609.}	43.2235	0.213383	newly	116.	110.184	{52., 221.}	44.2604	0.208246	Reff	0.957197	0.583005	{0.513699, 0.642384}	39.0925	ar	4824.	7059.45	{6936., 7191.}	46.3401
33	R0	{1.35084, 6.08841}	{2.45111, 18.3984}	{{2.35866, 16.9062}, {2.46477, 18.9032}}	{81.4504, 202.188}	inf	251.	1519.41	{970., 2184.}	506.913	0.764813	newly	266.	541.155	{323., 776.}	105.962	0.299779	Reff	0.778759	0.648902	{0.392481, 1.01203}	40.0211	ar	4759.	8861.68	{8708., 9075.}	86.2088
34	R0	{1.36912, 6.316}	{1.9258, 12.0848}	{{1.88607, 11.9453}, {1.94282, 12.1446}}	{40.6593, 91.3364}	inf	243.	711.283	{311., 1173.}	192.833	0.420374	newly	247.	379.35	{166., 684.}	67.996	0.207163	Reff	0.921965	0.62646	{0.4655, 0.849595}	37.5753	ar	4537.	7764.35	{7594., 7888.}	71.134
35	R0	{1.35831, 7.09038}	{2.20653, 13.9993}	{{2.18932, 13.5121}, {2.21411, 14.2212}}	{62.4468, 97.4414}	inf	210.	715.606	{329., 1272.}	240.765	0.47903	newly	184.	319.727	{146., 573.}	83.7615	0.231489	Reff	0.809553	0.477	{0.381197, 0.560337}	45.5864	ar	4667.	8454.23	{8359., 8558.}	81.1491
36	R0	{1.28315, 5.89938}	{2.28607, 13.096}	{{2.18735, 12.1087}, {2.31231, 13.3585}}	{78.1607, 121.99}	inf	195.	854.841	{419., 1480.}	338.38	0.592652	newly	206.	409.508	{195., 704.}	102.797	0.258077	Reff	0.765399	0.478258	{0.358408, 0.550337}	44.1377	ar	4460.	8600.75	{8413., 8720.}	92.8418
37	R0	{1.19303, 2.75113}	{1.69756, 6.15907}	{{1.69756, 6.15907}, {1.69756, 6.15907}}	{42.2898, 123.874}	inf	59.	252.525	{68., 559.}	329.28	0.513416	newly	124.	233.025	{63., 533.}	113.286	0.289558	Reff	0.868407	0.582777	{0.518944, 0.648807}	32.8956	ar	3142.	6932.93	{6787., 7053.}	120.653
38	R0	{1.36033, 7.8088}	{2.11408, 12.9748}	{{2.11292, 12.9719}, {2.11292, 12.9719}}	{55.4092, 66.1565}	inf	289.	778.926	{344., 1487.}	171.383	0.383289	newly	222.	374.13	{165., 737.}	80.7558	0.232329	Reff	0.859593	0.50575	{0.406526, 0.701067}	41.164	ar	4941.	8272.67	{8181., 8388.}	67.429
39	R0	{1.27353, 3.70845}	{1.96658, 10.2409}	{{1.96658, 10.2409}, {1.96658, 10.2409}}	{54.4196, 176.15}	inf	54.	511.386	{267., 756.}	847.012	0.931716	newly	83.	297.909	{145., 460.}	258.927	0.50685	Reff	0.815441	0.534798	{0.45074, 0.606297}	40.0118	ar	3725.	7899.32	{7807., 8017.}	112.062
40	R0	{1.19983, 12.1486}	{1.59433, 19.1171}	{{1.59433, 19.1171}, {1.59433, 19.1171}}	{32.8797, 57.3605}	inf	143.	432.2	{229., 622.}	202.844	0.446332	newly	81.	151.4	{61., 217.}	93.0041	0.26146	Reff	0.938027	0.747608	{0.597395, 0.856315}	26.4238	ar	3619.	6407.07	{6165., 6603.}	77.0397
41	R0	{1.20935, 2.67169}	{1.74755, 6.03899}	{{1.7459, 6.01426}, {1.7459, 6.01426}}	{44.5035, 126.036}	inf	33.	221.263	{101., 330.}	570.494	0.763896	newly	73.	201.053	{94., 302.}	176.64	0.376728	Reff	0.803008	0.549288	{0.502295, 0.593606}	31.5962	ar	3429.	7113.71	{6947., 7271.}	107.457
42	R0	{1.23313, 4.74781}	{1.86902, 12.6353}	{{1.86902, 12.6353}, {1.86902, 12.6353}}	{51.5672, 166.129}	inf	154.	629.133	{286., 891.}	308.528	0.565227	newly	207.	316.844	{133., 463.}	68.438	0.218558	Reff	0.853203	0.59547	{0.495477, 0.689295}	33.0098	ar	3835.	7602.69	{7490., 7706.}	98.2448
43	R0	{1.26105, 2.39713}	{1.95297, 8.87593}	{{1.95355, 8.88568}, {1.95355, 8.88568}}	{54.8685, 270.273}	inf	51.	523.696	{220., 881.}	926.854	0.954809	newly	120.	347.804	{143., 595.}	190.598	0.399218	Reff	0.808711	0.529715	{0.447363, 0.597005}	37.2417	ar	3723.	7856.09	{7744., 7950.}	111.015
44	R0	{1.36948, 8.88006}	{1.88607, 11.9453}	{{1.88607, 11.9453}, {1.88607, 11.9453}}	{37.7216, 34.5182}	inf	313.	825.795	{484., 1224.}	166.998	0.403418	newly	232.	444.909	{243., 688.}	96.0619	0.269705	Reff	0.929192	0.73372	{0.517726, 0.968686}	33.0087	ar	4437.	7626.95	{7442., 7762.}	71.8944
45	R0	{1.34882, 3.3791}	{2.13144, 8.28101}	{{2.12684, 8.23415}, {2.13505, 8.31782}}	{58.0224, 145.065}	inf	128.	657.109	{322., 1204.}	413.367	0.656957	newly	208.	460.078	{219., 935.}	123.625	0.293318	Reff	0.796074	0.486856	{0.387512, 0.622794}	42.1133	ar	4422.	8312.84	{8172., 8413.}	87.9883
46	R0	{1.23774, 4.34917}	{1.92454, 9.27343}	{{1.93462, 9.38215}, {1.93462, 9.38215}}	{55.4885, 113.223}	inf	149.	682.455	{330., 1109.}	358.023	0.624141	newly	217.	451.159	{192., 757.}	112.662	0.294793	Reff	0.844634	0.593233	{0.475336, 0.743668}	32.332	ar	3685.	7730.68	{7488., 7920.}	109.788
47	R0	{1.34327, 4.78811}	{2.30856, 13.321}	{{2.31231, 13.3585}, {2.31231, 13.3585}}	{71.8613, 178.21}	inf	220.	1456.93	{891., 1976.}	562.239	0.795918	newly	304.	699.37	{396., 1039.}	132.103	0.343336	Reff	0.872454	0.682067	{0.438876, 1.07869}	35.6293	ar	4517.	8636.91	{8537., 8732.}	91.2089
48	R0	{1.24366, 4.25814}	{1.88855, 11.0509}	{{1.88025, 10.9611}, {1.91302, 11.3733}}	{51.8544, 159.525}	inf	141.	739.477	{446., 1106.}	424.452	0.687723	newly	200.	424.773	{267., 630.}	115.318	0.308213	Reff	0.862976	0.66701	{0.518573, 0.831447}	31.8555	ar	3678.	7635.05	{7517., 7769.}	107.587
49	R0	{1.36017, 7.5415}	{1.6568, 10.5289}	{{1.6568, 10.5289}, {1.6568, 10.5289}}	{21.8083, 39.6128}	inf	178.	406.541	{164., 818.}	135.591	0.329858	newly	164.	248.135	{93., 509.}	74.4067	0.236793	Reff	1.01999	0.673409	{0.560661, 0.825086}	35.3919	ar	4178.	6716.49	{6572., 6880.}	60.7584
50	R0	{1.25333, 6.63578}	{1.67732, 9.44073}	{{1.67732, 9.44073}, {1.67732, 9.44073}}	{33.8291, 42.2701}	inf	109.	181.395	{62., 353.}	84.1864	0.241953	newly	93.	117.184	{36., 242.}	61.8846	0.232501	Reff	0.878334	0.578976	{0.528859, 0.625976}	34.0825	ar	3401.	6805.08	{6638., 6961.}	100.091
